# Ostreid herpesvirus 1 detection and relationship with *Crassostrea gigas *spat mortality in France between 1998 and 2006

**DOI:** 10.1186/1297-9716-42-73

**Published:** 2011-06-02

**Authors:** Céline Garcia, Anne Thébault, Lionel Dégremont, Isabelle Arzul, Laurence Miossec, Maeva Robert, Bruno Chollet, Cyrille François, Jean-Pierre Joly, Sylvie Ferrand, Nolwenn Kerdudou, Tristan Renault

**Affiliations:** 1 Institut Français pour la Recherche et l'Exploitation de la MER (IFREMER), Laboratoire de Génétique et de Pathologie, Ronce les Bains, 17390 La Tremblade, France; 2 Agence Française de Sécurité Sanitaire des Aliments (AFSSA), PASER-DERNS, 27-31 Avenue du General Leclerc, 94701 Maisons-Alfort, France; 3 Institut Français pour la Recherche et l'Exploitation de la MER (IFREMER), Service Valorisation de l'Information pour la Gestion Intégrée et la Surveillance, rue de l'île d'Yeu, BP 21105, 44311 Nantes Cedex 03, France

## Abstract

Since its molecular characterisation, Ostreid herpesvirus 1 (OsHV-1) has been regularly detected in *Crassostrea gigas *in France. Although its pathogenicity was demonstrated on larval stages, its involvement during mortality outbreaks at the juvenile stage was highly suspected but not evidenced. To investigate mortality outbreaks, the French National Network for Surveillance and Monitoring of Mollusc Health (REPAMO) carried out two surveys in juvenile *C. gigas*. The first survey lasted from 1998 to 2006 and was an epidemiological inquiry occurring when oyster farmers reported mortality outbreaks. The second survey, a longitudinal one, was set up in 1998 to complete the network observations on OsHV-1. Data analysis showed a specific pattern of mortality outbreaks associated with OsHV-1 detection. Ostreid herpesvirus 1 detection mainly appeared during the summer, suggesting the influence of the seawater temperature on its occurrence. It mostly presented a patchy distribution in the field in contrast to the nursery. Significant relationship between OsHV-1 detection and spat mortality was found, preferentially in sheltered and closed environments. The longitudinal survey confirmed most of the network observations. Although subsequent works particularly epidemiological surveys would be useful to confirm the causal link between the detection of OsHV-1 and the mortality outbreaks in juvenile *C. gigas*, the role of OsHV-1 in oyster mortality is progressing.

## Introduction

In France, oyster production is mainly based on one species, the Pacific cupped oyster, *Crassostrea gigas*, which was introduced in the late 1960's and the early 1970's and replaced the Portuguese oyster, *C. angulata*, after its disappearance along the French coasts in relation to irido-like virus infections [1]. Since its introduction in France, sporadic mortalities of both spat and adult *C*. *gigas *have been regularly observed in the summertime along the French coasts [2]. Meanwhile, massive mortalities were reported on larvae and spat of *C. gigas *first in hatcheries and nurseries and secondly in the field between 1991 and 1995 [3]. Since these events, recurrent and seasonal losses have occurred in France, mainly during the summer period. Pacific oyster mortality outbreaks so called oyster summer mortalities were also reported in different parts of the world and may greatly affect *C. gigas *production. Oyster summer mortalities were reported for the first time in Japan in 1915 [4] and then in the USA in the late 1950's [5]. In most cases, summer mortalities have not been explained by a single factor but rather by the combination of several parameters including physiological stress, environmental conditions and pathogenic organisms; notably, relationships between gonadal maturation, energy metabolism and mortalities were investigated in different countries and correlations were shown between the poor quantity of glycogen in spat, reproductive effort and mortality [2,6-9].

During the French 1991-1995 mortality outbreaks, a virus was detected associated with mortalities and assigned as a member of the Herpesviridae family [10,11]. The first description of herpes-like virus associated with mollusc mortality was reported in 1972 in adult oysters *Crassostrea virginica *of the east coast of the USA [12]. Since this date, herpes-like viruses have been associated with significant mortality rates in other mollusc species such as oysters [11,13-16], scallops [17], clams [18] and more recently abalones [19,20]. One of these viruses collected during a mortality outbreak of French Pacific oyster larvae in 1995 was first characterised as a member of the *Herpesviridae *family and named *Ostreid herpesvirus 1 *(OsHV-1) [21,22]. Recently, this virus was reclassified in the order of *Herpesvirales *as the unique member of the family of *Malacoherpesviridae *[23].

Transmission experiments of the disease to axenic *Crassostrea gigas *larvae demonstrated the pathogenicity of the virus for Pacific oyster larvae [24] and they also indicated that OsHV-1 is responsible for infections observed in larvae belonging to different bivalve species [25]. Mortality of juvenile Pacific oysters *C. gigas *and European flat oysters, *Ostrea edulis *has also been associated with herpes-like viruses [3,14,26-28] but the ability of such a virus to kill juvenile oysters was not truly demonstrated [29].

Demonstrating the causal link between a pathogenic agent and mortalities is not only based on the ability to reproduce the disease but it must incorporate the idea of multiple causal factors. The development of a set of shaping principles to guide evaluation of theories of disease causation was started by Koch [30] and refined then by Hill and Evans [31,32]. Hill and Evans formulated a list of postulates that are widely accepted guidelines for evaluating evidence of causation in epidemiology. In the process to better understand oyster mortality outbreaks in France, the National Network for Surveillance and Monitoring of Mollusc Health called REPAMO (REseau de PAthologie des MOllusques) carried out both a systematic research of infectious agents including OsHV-1 and an epidemiological investigation when cases of *C. gigas *spat abnormal mortality were reported in France. This mainly passive surveillance was based on oyster farmer declarations and OsHV-1 detection was performed by PCR which has been described as a technique of choice for detecting this virus in larvae and spat [33]. In parallel, a longitudinal survey, based on an active surveillance, was performed during the summer 1998 in one particular site in order to complete the network observations. The objective was to characterise oyster spat mortality in this site, to estimate the mortality level, and to establish the role of OsHV-1 on the reported mortality events.

This paper reports the synthesis of nine years from 1998 to 2006 of surveillance performed by the REPAMO network as well as the characteristics of mortality events associated with OsHV-1 detection in spat of *Crassostrea gigas *in France. It also discusses the implication of OsHV-1 in oyster spat mortality.

## Materials and methods

### Sampling strategy

#### REPAMO network surveillance

##### • Data from passive surveillance

Abnormal mortality in mollusc aquaculture was defined in the European Directive 95/70/EC [34] as a sudden mortality affecting approximately 15% of stocks and occurring over a short period between two inspections (confirmed within 15 days). In practice, the study of abnormal mortality outbreaks in France is the subject of a passive surveillance. Abnormal mollusc mortality cases are reported by farmers themselves to the competent authority (Maritim Affairs). The REPAMO network records reported mortality cases, collects representative samples of these mortality cases and performs analyses on these samples. For each mortality case, an epidemiological investigation is carried out with farmers by face to face interviewing using a questionnaire in order to collect information and explore the different factors that could be involved in the reported mortality events. This questionnaire is composed of different parts including questions about the animal sampled (origin, species, age, history including previous transfers, tide coefficient of leasing ground), the abnormal mortality event (date, description, site, affected species, determination of mortality rate), the growing methods (on bottom, racks, cages...) and the environmental parameters.

Between 1998 and 2006, 200 and 62 samples of *Crassostrea gigas *spat (younger than one year old) were collected in the field and in inland facilities (nursery) respectively during abnormal mortality events representing a total of 10 816 individuals. These samples came from the main oyster production areas (Normandy, North and South Brittany, Vendée, Charente-Maritime, Arcachon bay, Mediterranean lagoons). Collected oysters were mainly chosen among moribund individuals meaning individuals still alive but weakened. Among these 262 collected samples, 145 were investigated both by PCR and histology and 117 only by PCR. On average, for each mortality case, 30 individuals were analysed by histology and 30 by PCR (6 pools of 5 oysters with as far as possible, 3 pools composed of moribund oysters and 3 of live oysters).

##### • Data from active surveillance

Between 1998 and 2001, an active survey was performed by the REPAMO network in order to better know the OsHV-1 distribution before and during the critical period of summer mortality of *Crassostrea gigas *spat in different production areas (Mediterranean lagoons, Charente-Maritime, Vendée, South Brittany). For each sampling site, three oyster bags were chosen and dead and live oysters contained in one liter volume of each bag were counted; mortality estimation was determined based on the average of the counting of the three oyster bags.

During this period, 358 samples (25 individuals per sample) were collected monthly then weekly during the critical period and only OsHV-1 PCR analyses were performed on the animals sampled.

#### Longitudinal survey

In 1998, a survey was implemented in the site of Pointe de la Fumée (Charente-Maritime, France) which counted 156 leasing grounds belonging to 117 oyster farms. This site was chosen according to different factors: (i) severe *Crassostrea gigas *spat mortality outbreaks occurred in 1994 and 1995 for which OsHV-1 was detected, (ii) it is a traditional site of wild catch spat production for other French areas, (iii) spat are collected and kept in place for growing, (iv) rearing methods are standardised between oyster farms and (v) oyster spat can be easily collected every 15 days. For the pre-study in 1997, the local competent authority gave the name and address of each breeder of each lease of the site. Thirty breeders were randomly selected from the list using the RAND function of Excel software and asked for a questionnaire. Only eight of them were keeping some spat during the whole summer period and agreed to the study.

Twelve leasing grounds belonging to the 8 farms were randomly chosen in this site for the survey in 1998 and three bags of around 1000 wild caught spat (10 mm size) were followed in each leasing ground. Between May 1^st ^and September 8^th^, once a month in May and June and every two weeks in July and August, 30 live spat per bag were collected for OsHV-1 PCR analysis and mortality estimation was determined on each oyster bag by counting the dead and live oysters contained in one litre volume. Water temperature was measured every 15 min with a YSI^® ^probe.

### Sample analysis

#### Histology

A slice of tissues of each animal including most of the organs like digestive gland, mantle, gonad, gills, labial palps and kidney was fixed in Davidson's fixative for 48 h, dehydrated and embedded in paraffin. One section per individual (2 to 3 μm thick) was cut and stained with haematoxylin-eosin for examination.

#### Animal sample preparation and OsHV-1 PCR analysis

Analyses were performed according to the methods described previously [33,35]. All analyses were performed on pools of animals and each pool contained five animals. Animals lesser than 12 mm were pooled in tubes, weighed, grounded adding double distilled water. For spat larger than 12 mm, animals were removed from the shell, dried, pooled in plastic bags, grounded with a rubber mallet and 0.5 g oyster tissues were removed and 1 mL of double distilled water was added. Spat homogenates were incubated in boiling water bath for 10 min, then mixed and centrifuged at 9000 *g *for 5 min. Samples were analysed using the primer pair C2 (CTCTTTACCATGAAGATACCCACC) and C6 (GTGCACGGCTTACCATTTTT) and each 50 μL PCR reaction contained the appropriate reaction buffer (10 mM Tris, pH 8.3; 50 mM KCl), 0.05 mM of each dNTP, 100 ng of each primer, 2.5 mM MgCl_2_, 2.5 U of DNA polymerase Goldstar (Eurogentec, Brussels, Belgium) and 1 μL of template DNA. Amplifications were performed with an initial denaturation step at 94°C followed by 35 cycles at 94°C for 1 min, 50°C for 1 min and 72°C for 1 min with a final elongation at 72°C for 5 min. PCR products were separated on 1% agarose gel containing ethidium bromide (0.1 μg/mL) and visualised using a UV transilluminator. The size of the expected PCR products was 709 bp. A sample is considered as positive when a pool is positive.

During each PCR experiment, negative and positive controls were included; the presence of potential PCR inhibitors has been systemically investigated using an internal standard [36] when PCR results were negative.

### Data analysis

#### Surveillance network data

##### Variable definitions

The OsHV-1 detection is the number of OsHV-1 positive samples. Months included March to November since no sample animal was collected from December to February. Tide influence was appreciated by tide coefficients divided into three categories: low (< 50), medium (> 50 and < 70) and high (> 70). The stressful effects were estimated by farmers and defined as a sudden environmental change (weather changes as strong rains, storm, hot sunny period, phytoplanctonic bloom and visible pollution) or a change of rearing conditions (transfer, oyster handling, spat removing from collectors) just before mortality observations (less than one week); these data are subjective observations. Site typology was defined by four categories: inland facilities (nursery), closed environments such as a lagoon, semi-closed environments such as an estuary and open environments. The mortality rate has been appreciated by the average percentage of mortality obtained by counting three oyster bags. The characteristics of the mortality were defined by its pattern (patchy, uniform or concentric) and its development (acute or chronic).

##### Statistical procedures used

*• Analysis of link between OsHV-1 and other factors*

Data from passive and active surveillance were used for these analyses.

The GENMOD procedure of the SAS statistical software package version 9.1 using a binomial distribution and a probit link function was performed in order to identify which parameters including months, tide influence, stressful effects and site typology were associated with the OsHV-1 detection.

*• Analysis of link between mortality and OsHV-1*

Data from passive surveillance were used for these analyses.

A χ^2 ^test [37] was performed using SAS software version 9.1 to compare the OsHV-1 detection when samples were either composed with only live oysters or moribund and live oysters. The same approach was performed to compare OsHV-1 detection when sampling events occurred within the week following the onset of mortality or later.

Percentages of mortality were analysed by generalised linear mixed effect (probit link) (GLMM) using the GLIMMIX procedure with SAS software. Parameters of the GLMM were fitted with Laplacian approximation [38]. The likehood-ratio test and the Akaike information criterion (AIC) index were used to compare nested and non nested models of GLMM respectively [39]. OsHV-1 detection and month were considered as fixed effects while site, year and their interaction were used as random effects.

The GLIMMIX procedure was also used to describe the typology of mortality (random effect) associated to OsHV-1 detection within field or inland facilities.

##### Data analysis for longitudinal monitoring of spat during the summer 1998

Globally the same statistical analysis was performed as reported above except that because 3 bags of oysters were clustered in the same leasing ground and the same bags and repeated observations were made on the sampling site, responses were likely to be correlated, so, an autoregressive correlation was specified in the GENMOD procedure AR(1) [40] and the effect of the bags inside the same leasing ground was taken as a random effect in the lme4 procedure.

The temperature was determined as the mean over 24 h.

## Results

### Network surveillance

#### OsHV-1 detection (data from passive and active surveillance)

From 1998 to 2006, no relevant pathogen agent was detected in histology in *Crassostrea gigas *spat. Abnormal tissue architecture and cellular changes were noted for 72% of the sampling events with an average of 44% of individuals concerned per sample. The main lesions were haemocyte infiltrations, mostly in connective tissues of gills and the digestive gland and nucleus abnormalities in the connective tissues of different organs mainly characterised by the presence of pycnotic nuclei and margined chromatin.

For OsHV-1 detection by PCR, detectable amplicons were reported in 18% of the 620 samples screened, representing 93 and 21 sampling events from the passive and active surveillance respectively (Table 1). All PCR negative control reactions gave negative results whereas positive control reactions as well as reactions involving the internal standard gave positive results. OsVH-1 detection was significantly increased when either samples contained moribund oysters or sampling occurred within a week following the onset of mortality (*p *< 0.001) (Table 2).

**Table 1:** *Crassostrea gigas *samples collected during passive and active surveillance in France between 1998 and 2006: OsHV-1 detection results by PCR.

Year	Passive surveillance samples				Active surveillance samples			
	Number of samples	Number of OsHV-1 negative samples	Number of OsHV-1 positive samples	OsHV-1 detection frequency (%)	Number of samples	Number of OsHV-1 negative samples	Number of OsHV-1 positive samples	OsHV-1 detection frequency (%)
1998	32	29	3	9	120	110	10	8
1999	19	15	4	21	102	98	4	4
2000	38	30	8	21	48	44	4	8
2001	53	33	20	38	88	85	3	3
2002	34	12	22	65	-	-	-	-
2003	23	12	11	48	-	-	-	-
2004	30	19	11	37	-	-	-	-
2005	17	7	10	59	-	-	-	-
2006	16	12	4	25	-	-	-	-
Total	262	169	93	35	358	337	21	6

**Table 2:** OsHV-1 PCR results (# of samples and %) according to the presence or absence of moribund oysters in the diagnosed sample and the onset of mortality.

OsHV-1 PCR results	Diagnosed sample		Onset of mortality	
	Presence of moribund oysters	Absence of moribund oysters	Less than one week	Greater than one week
Positive	78-70%	36-7%	74-52%	19-16%
Negative	34-30%	472-93%	67-48%	102-84%
Total	112	508	141	121

#### OsHV-1 characteristics (data from passive and active surveillance)

**Figure 1:**
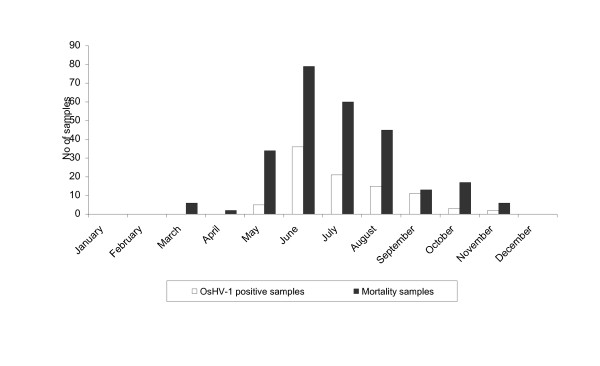
**Monthly number of both *Crassostrea gigas *spat samples collected for mortality and OsHV-1 positive samples among these samples during the years 1998 to 2006**.

OsHV-1 was not detected in March and April while it was from May to November (Figure 1). However, its detection frequency was significantly higher from June to September than the other months (*p *< 0.05).

**Figure 2:**
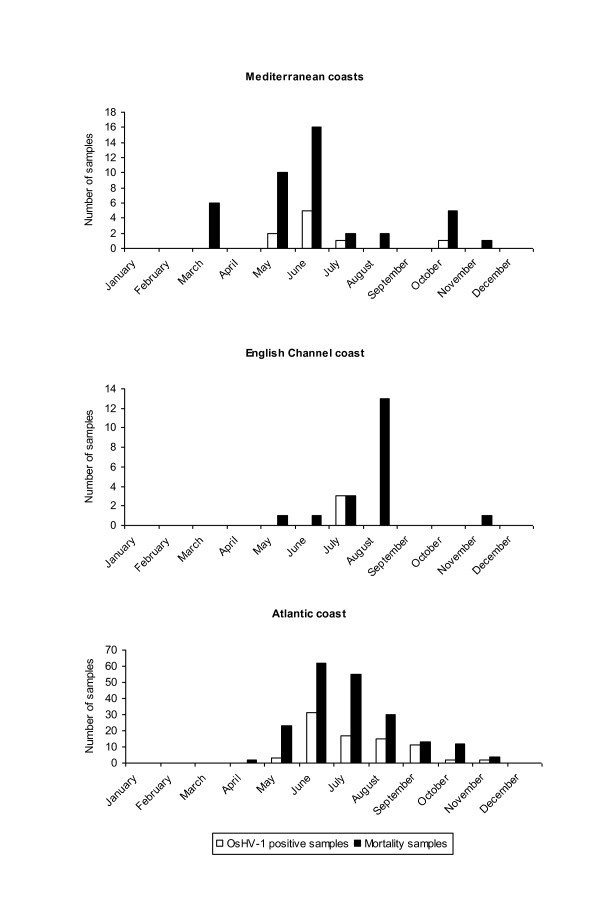
**Monthly number of both *Crassostrea gigas *spat samples collected for mortality according to the French coasts and OsHV-1 positive samples among these samples during the years 1998 to 2006**.

**Figure 3:**
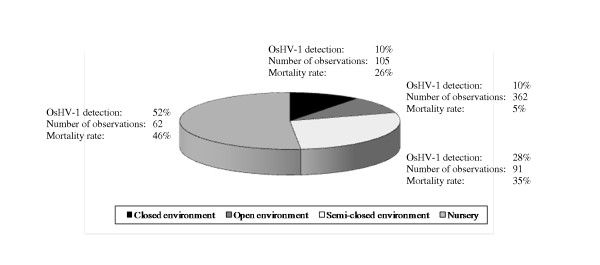
**Relative proportions of the total number of OsHV-1 positive samples of *Crassostrea gigas *and mortality rate between 1998 and 2006 according to the area typologies**.

The virus was detected in all growing areas where *C. gigas *is cultivated. It was mainly detected in sampling sites of Vendée, South Brittany and Charente-Maritime with respectively 35, 22 and 18% of positive results (Table 3). OsVH-1 detection was usually first reported in animals collected in the south of France (Mediterranean coasts) in later May, then in oyster spat collected along the Atlantic coast in June-July and finally in animals from the English Channel in July-August (Figure 2). OsHV-1 was detected more frequently in environments such as inland facilities (nursery) and semi-closed environments (estuary and rivers) with 52 and 28% of positive samples, respectively (*p *< 0.05) (Figure 3). The tide height was not a significant factor influencing OsHV-1 detection in oyster spat (*p *> 0.05), while it had a significant impact on spat which experienced stressful conditions before mortality observation (*p *< 0.001) (data not shown).

**Table 3:** Main mortality rate of *Crassostrea gigas *in the different French growing areas between 1998 and 2006 (passive surveillance) and proportions of OsHV-1 positive samples in each area (passive and active surveillance).

	Mediterranean lagoons	Arcachon Bay	Charente-Maritime	Vendée	South Brittany	North Brittany	Normandy
Mean mortality rate	41%	39%	37%	38%	43%	40%	50%
Mean OsHV-1 detection	18%	5%	18%	35%	22%	14%	14%

#### Abnormal mortality and OsHV-1 detection (data from passive surveillance)

During spat mortality outbreaks occurring from 1998 to 2006, the mean frequency of OsHV-1 detection was 35% and varied from 9 to 65% according to the year. For animals sampled when no mortality was reported, its annual mean frequency was 6% (Table 1). Virus detection generally coincided with the beginning of oyster spat mortality and it was no longer detected once spat mortality dropped.

The kinetics of OsHV-1 detection presented a similar pattern to the pattern reported for oyster spat mortality outbreaks (Figure 1) and these patterns were generally observed every year. For each geographical area, OsHV-1 detection peak mainly corresponded to the maximum reported mortality cases; this peak was reached at different time points according to the areas with a temporal progression from the south to the north of France (Figure 2).

The effects of month and OsHV-1 detection were always significant in relation with mortality rate (*p *< 0.0001), taking into account correlation inside site or in the same year and same site.

Significant differences of mortality characteristics were observed for the spat mortality in the field associated with OsHV-1 detection (*p *< 0.001) with mainly a patchy distribution and a rapid development (Table 4). In contrast, a uniform distribution of mortality was observed in nurseries, but it was mainly rapid (*p *< 0.001) (Table 4).

**Table 4:** Repartition of OsHV-1 negative and positive samples according to mortality characteristics in the field (A) or nursery (B) (*p < 0.001; ns: non-significant)

a					
	Field samples				
OsHV-1 PCR results	Mortality pattern*		Mortality development		
	Patchy	Uniform	Concentric	Acute	Chronic
Positive	44	14	0	56	2
Negative	42	92	8	120	22
Total	86	106	8	176	24

**b**					

	**Nursery samples**				
**OsHV-1 PCR results**	**Mortality pattern**^ns^		Mortality development*		
	**Patchy**	**Uniform**	**Concentric**	**Acute**	**Chronic**

Positive	5	30	0	34	1
Negative	3	24	0	15	12
Total	8	54	0	49	13

### Longitudinal survey

**Figure 4:**
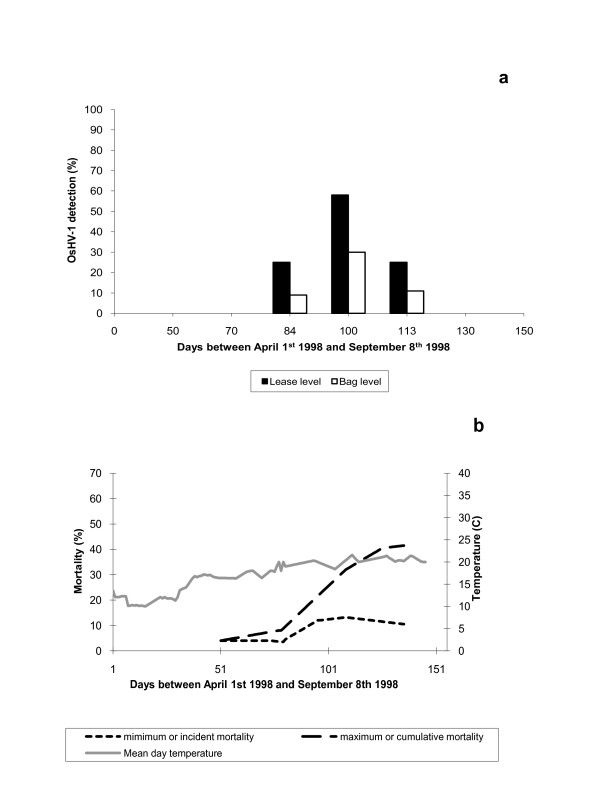
**OsHV-1 detection (a) and mortality rate (b) associated at lease and oyster bag level during the 1998 survey**.

During the period studied, mortality was minimal until late June. Recent mortalities were reported at the end of June and reached a maximum in July to decrease in August (Figure 4). The average cumulative mortality at the end of the survey was 30% and it was significantly different between leasing grounds and between periods of sampling (*p *< 0.05). Temperature influence was also noted; mortality seemed to appear with the increasing of the mean day temperatures.

OsHV-1 was detected in 18 oyster spat samples among the 200 analysed samples. All the PCR negative control reactions gave negative results whereas positive control reactions as well as reactions involving the internal standard gave positive results.

**Figure 5:**
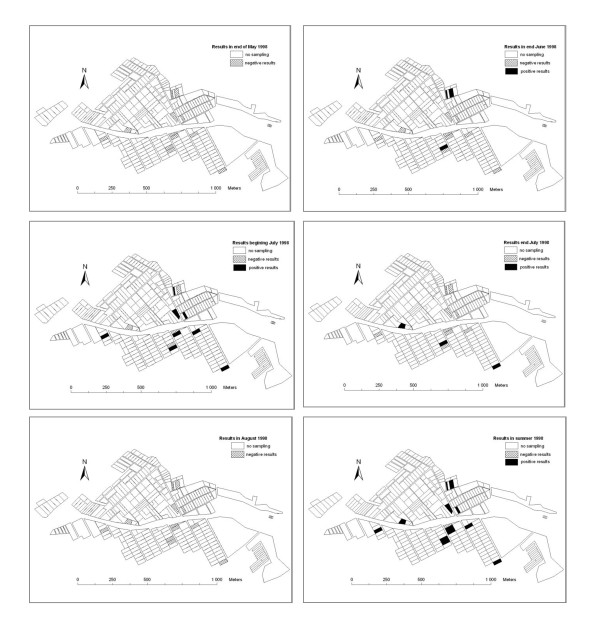
**Leases sampled during the 1998 survey and OsHV-1 detection and distribution among leases between May and August 1998**.

The first detection of OsHV-1 was at the end of June and it reached a peak at the beginning of July (Figures 4 and Figure 5). OsHV-1 was no longer detected at the end of August and at the end of the study, OsHV-1 was detected in all leasing grounds except one (Figure 5). Virus detection was not systematic in the three bags of one leasing ground (generally 1 positive bag per leasing ground) but mainly presented a patchy distribution between bags and also between leasing grounds (Figure 5). OsHV-1 was detected just before mortalities began and its detection continued until the mortality peak was reached (Figure 4). The overall mortalities from May to August were not correlated with OsHV-1 detection; however, a significant correlation was found between the detection of virus and the beginning of mortalities (*p *< 0.05).

## Discussion

Very few epidemiological studies have been carried out in order to elucidate the implication of OsHV-1 in Pacific oyster spat mortality. For oyster larvae, it is well established that OsHV-1 induced larval mortalities [24,35] and a causal link between OsHV-1 presence and larval mortality has been demonstrated [41]. For oyster spat, this link appears more difficult to establish, probably due to major interactions with other factors. A longitudinal survey was set up in the west coast of the USA and suggested the implication of OsHV-1 as a potential factor of oyster spat mortalities but further investigations are needed to conclude on this point [28]. Throughout both surveys reported in the present study, many data were collected on OsVH-1 detection and spat mortality during several years. Data permitted to draw a pattern of OsHV-1 detection during oyster spat mortality events despite the fact that the majority of the reported data were issued from a passive surveillance. One of the consequences of passive surveillance could be the under declaration of mortality events and an estimation of certain parameters (mortality rate, mortality date, environmental parameters) [42,43]. Meanwhile, the longitudinal survey reinforced some results obtained through the surveillance network.

In the present work, OsHV-1 was regularly detected when oyster spat mortality events occurred both in the field and in nurseries. No other relevant pathogenic agent except bacteria belonging to the *Vibrio *family was detected. Classical bacteriology has been systematically performed on mortality cases since 2002 (data not shown) and some virulent *Vibrio *strains (*Vibrio aestuarianus*, *V. splendidus *and *V. harveyi*) were detected in some mortality events [44]. Meanwhile, only five reported mortality cases presented a co-detection of OsHV-1 and *Vibrio *strains since 2002. Considering this low number of co-detection, the impact of bacteria was not taken into account in the present study on OsHV-1.

Ostreid herpesvirus 1 was significantly detected during the summer which might suggest a link between seawater temperature and OsHV-1 infection. The virus was first detected in southern France where temperatures became rapidly high and its detection followed a gradient of increasing temperatures along the French coast. In Fouras (Charente-Maritime), OsHV-1 detection was reported when an increase of mean day temperature occurred rapidly. A similar pattern was noted for mortality appearance. During inquiries, oyster farmers often mentioned an increase in seawater temperature before the mortality began. Indeed, OsHV-1 was often detected when temperatures increased quickly and it was no longer detected once temperatures were stable albeit remaining high. Similar observations have been reported in the field for oyster spat in Tomales Bay (USA) and in experimental conditions (Ifremer, La Tremblade, France): mortality occurred after a temperature spike [28,45]. The temperature influence on OsHV-1 detection and virus expression was demonstrated for *Crassostrea gigas *larvae [46] and strongly suspected for *C. gigas *spat [27,45,47,48]. Although the present work showed similar tendencies, a temperature threshold related to enhanced OsHV-1 expression or mortality appearance appears difficult to define precisely. In the literature, according to the site, the temperature threshold was variable: 22°C to 25°C in the west coast of the USA [27,48] and 18 to 20°C in France [9,49]. High seawater temperatures appear as one of the potential factors inducing OsHV-1 infection as observed with the Koi herpesvirus where temperatures between 18 and 28°C favoured the onset and the severity of the disease in fish [50,51]. The question of temperature influence (is it a rapid increase of seawater temperature or the reach of a minimum temperature threshold) on the onset of the disease remains open. Moreover, stressful conditions particularly rearing techniques seem to favour OsHV-1 infection as reported in the present study and by Renault and Novoa [29]. Similar results have been reported for other members of Herpesviridales [52]. In France, during the summer, many oyster transfers occur and these transfers are a source of stress for oysters (environment change, handling, transport) and thus may also amplify OsHV-1 detection. So, the temperature increase and cultural practices may favour OsHV-1 detection during the summer and could affect the onset of the disease.

Spat mortality outbreaks associated with OsVH-1 detection generally presented a patchy distribution in the field. This particular pattern could be partly explained by the nature of the virus. Herpesviruses are enveloped and assumed to present a weak resistance in their environment. Thus, their transmission relies generally on direct contact [52]. Ostreid herpesvirus 1 can be detected in seawater but its detection appears limited in time, the temperature being one of the most important factors [45,53]. Moreover, marine waters may contain various substances such as nucleases or antiviral factors that could destroy the virus or inhibit its activity [54]. These data suggest that when OsHV-1 is excreted by oysters, it can mainly infect close oysters. This phenomenon was also observed during the longitudinal survey in Fouras: in one leasing ground, one oyster bag could be infected by OsHV-1 and not the other ones. In a bag, oysters were very close and OsHV-1 transmission might be easier. The probable limited dissemination of OsVH-1 in seawater could partly explain the observation of the patchy mortality distribution rather than a uniform distribution as observed in nurseries. In nurseries, oysters are reared at high density, very close together and the seawater is often sequentially renewed. Virus dispersion and dilution may be important in the field due to the marine currents, hence the higher OsVH-1 detection in sheltered environments such as estuaries.

The hypothesis that OsHV-1 persistence is low in seawater can raise a question about the effective ways of oyster contamination occurring in the field: either OsHV-1 is introduced in the same time as oysters or there are OsHV-1 reservoirs in the environment. Both hypotheses are not exclusive. The first suggestion is reinforced by the fact that the members of the *Herpesviridae *family are generally able to persist in their host in an asymptomatic way, with virus expression being associated to stressful conditions [52]. Viral DNA and protein have been previously detected in asymptomatic adult oysters suggesting this possibility [55]. Oysters can also play the role of reservoir for OsHV-1 transmission. However, other molluscs can also play this role. Indeed, OsHV-1 was detected in several mollusc bivalves such as *Ostrea edulis*, *Ruditapes philippinarum*, *Ruditapes decussatus*, *Pecten maximus *[17,25] and some of these molluscs share the same habitat as *Crassostrea gigas*. Finally, the virus could be present in the field in different marine species other than mollusc bivalves acting as potential sources of viruses [56].

In our study, a relationship between OsHV-1 detection and oyster mortality was demonstrated. An increase in OsHV-1 detection was significantly related to an increase in mortality rate. Based on Hill and Evans criteria [32], [31], a causal link is not totally defined but several elements emphasize it. Ostreid herpesvirus 1 was detected in different parts of the world [27,29,57] generally associated with *Crassostrea gigas *spat mortality outbreaks. Positive significant correlations between spat mortality events and OsHV-1 detection were reported in the present study as in Tomales Bay in the USA [27,28]. Although specific macroscopic signs associated with OsHV-1 infection except bivalve mortality are missing due to the inexpressive nature of mollusc bivalves, histology examination demonstrates specific nucleus alterations (chromatin margination, nucleus hypertrophy) in infected animals as described by Renault et al. [11]. In the longitudinal survey, it was demonstrated that OsHV-1 was detected just before the beginning of oyster spat mortality and the virus DNA amounts increased during spat mortality as observed in the USA [28] and in a French area [58]. The criteria of consistency, specificity, temporality, plausibility, coherence and analogy proposed by Evans and Hill [32] were fulfilled. Meanwhile, the analysis of the causal link also revealed a lack of data and the need for epidemiological surveys. Indeed, no study has been developed to measure the strength of association or to evaluate the effectiveness of controlled measures or eradication regarding OsHV-1. It would be interesting to study the risk factors associated to OsHV-1 expression by carrying out a follow-up before, during and after mortalities with frequent animal sampling and to show the eventual role of human intervention on OsHV-1 infection in order to assign it as the cause of oyster spat death. Indeed, in our study, the mortality rate was found to be significantly different between leasing grounds in Fouras emphasizing differences of cultural practices between farmers, some practices decreasing or increasing mortality rate. The availability of new diagnostic tools like real time PCR [58] will also help determine the minimum OsHV-1 quantity associated with the observation of spat mortality.

In conclusion, this study brought different arguments to reinforce the causal link between OsHV-1 detection and spat mortality but also highlighted the need for epidemiological surveys. Our results suggest two measures for improving OsVH-1 detection during oyster mortality outbreaks: (1) collecting animals preferentially less than one week after mortality events and (2) carrying OsHV-1 PCR on both live and moribund oysters. This study also presented the originality to be one of the first studies to report the use of molecular tools to detect a mollusc pathogen organism over such a long period (nine years) and could also serve for studying the new mortality oubreaks occurring in France since 2008. Indeed, since the summer of 2008, severe mortality events in cultured Pacific oyster spat have been reported. The pattern of the mortality notifications seemed to differ from other years, with a higher number of notifications and higher mortality intensity (up to 80-100% in some areas) [59,60]. OsHV-1, especially a newly described variant OsHV-1μvar was frequently detected in affected populations [61]. Several studies have begun on the OsHV-1μvar variant and our study could be used as a reference to compare OsHV-1 and its OsHV-1μvar variant.

## Competing interests

The authors declare that they have no competing interests.

## Authors' contributions

This study is the result of a collective work. All authors conceived this study, and participated in its design. CG, CF, LM, IA and AT carried out the development of the study with the help of others authors. CG, BC, MR, NK, SF and JPJ carried out histological and molecular analysis. LD, CG and AT performed the statistical analysis. CG drafted the manuscript with the help of TR, LD and AT. All authors read, corrected, and approved the final manuscript.

## References

[B1] Comps M Bonami JR Vago C Campillo A Une virose de l'huître portugaise (*Crassostrea angulata *Lmk) C R Hebd Séanc Acad Sci 1976 282 1991 (in French)

[B2] Maurer D Comps M His E Caractéristiques des mortalités estivales de l'huître *Crassostrea gigas *dans le bassin d'Arcachon Haliotis 1986 15 309 317 (in French)

[B3] Renault T Le Deuff RM Cochennec N Maffart P Herpesviruses associated with mortalities among Pacific oyster, *Crassostrea gigas*, in France - Comparative study Revue Méd Vét 1994 145 735 74219594077

[B4] Takeuchi T Takemoto Y Matsubara T Haematological study of bacterial affected oysters Rep Hirshima Prefect Fish Exp Stn 1960 22 1 7

[B5] Glude JB Summary report of Pacific coast oyster mortality investigations 1965-1972 Proceedings of the First U.S.-Japan meeting on Aquaculture 1975 1 28

[B6] Cotter E Malham SK O'Keeffe S Lynch SA Latchford JW King JW Beaumont AR Culloty SC Summer mortality of the Pacific oyster, *Crassostrea gigas*, in the Irish Sea: The influence of growth, biochemistry and gametogenesis Aquaculture 2010 303 8 21 10.1016/j.aquaculture.2010.02.030

[B7] Huvet A Normand J Fleury E Quillien V Fabioux C Boudry P Reproductive effort of Pacific oysters: A trait associated with susceptibility to summer mortality Aquaculture 2010 304 95 99 10.1016/j.aquaculture.2010.03.022

[B8] Numachi KI Oizumi J Sato S Imai T Studies on the mass mortality of the oyster in Matsushima Bay. III. The pathological changes of the oyster caused by Gram-positive bacteria and the frequency of their infection Bull Tohoku Reg Fish Res Lab 1965 25 39 48 (in Japonese)

[B9] Samain JF McCombie H Quae, Versailles Summer mortality of Pacific oyster *Crassostrea gigas* The Morest project 2008

[B10] Nicolas JL Comps M Cochennec N Herpes-like virus infecting Pacific oyster larvae, *Crassostrea gigas* Bull Eur Ass Fish Pathol 1992 12 11 13

[B11] Renault T Cochennec N Le Deuff RM Chollet B Herpes-like virus infecting Japanese oyster (*Crassostrea gigas*) spat Bull Eur Ass Fish Pathol 1994 14 64 66

[B12] Farley CA Banfield WG Kasnic JRG Foster WS Oyster herpes-type virus Science 1972 178 759 760 10.1126/science.178.4062.759 5082840

[B13] Hine PM Wesney B Hay B Herpesvirus associated with mortalities among hatchery-reared larval Pacific oysters *Crassostrea gigas* Dis Aquat Organ 1992 12 135 142

[B14] Comps M Cochennec N A herpes-like virus from the European oyster *Ostrea edulis* J Invertebr Pathol 1993 62 201 203 10.1006/jipa.1993.1098

[B15] Hine PM Thorne T Replication of herpes-like viruses in haemocytes of adult flat oysters *Ostrea angasi *(Sowerby 1871): an ultrastructural study Dis Aquat Organ 1997 29 197 204

[B16] Hine PM Wesney B Besant P Replication of herpes-like viruses in larvae of the flat oyster *Tiostrea chilensis *at ambient temperatures Dis Aquat Organ 1998 32 161 171

[B17] Arzul I Nicolas JL Davison AJ Renault T French scallops: A new host for Ostreid Herpesvirus-1 Virology 2001 290 342 349 10.1006/viro.2001.1186 11883198

[B18] Renault T Lipart C Arzul I A herpes-like virus infects a non-ostreid bivalve species: virus replication in *Ruditapes philippinarum *larvae Dis Aquat Organ 2001 45 1 7 11411639 10.3354/dao045001

[B19] Chang PH Kuo ST Lai SH Yang HS Ting YY Hsu CL Chen HC Herpes-like virus infection causing mortality of cultured abalone *Haliotis diversicolor supertexta *in Taiwan Dis Aquat Organ 2005 65 23 27 16042040 10.3354/dao065023

[B20] Tan J Lancaster M Hyatt A van Driel R Wong F Warner S Purification of a herpes-like virus from abalone (*Haliotis *spp.) with ganglioneuritis and detection by transmission electron microscopy J Virol Methods 2008 149 338 341 10.1016/j.jviromet.2007.12.019 18374425

[B21] Minson AC Davison A Eberle R Desrosiers RC Fleckstein B McGeoch DJ Pellet PE Roizman B Studdert DMJ Family Herpesviridae Virus Taxonomy, Seventh Report of the International Committee on Taxonomy of Viruses 2000 Academic Press, USA 203 225

[B22] Davison AJ Trus BL Cheng N Steven AC Watson MS Cunningham C Le Deuff RM Renault T A novel class of herpesvirus with bivalve hosts J Gen Virol 2005 86 41 53 10.1099/vir.0.80382-0 15604430

[B23] Davison AJ Eberle R Ehlers B Hayard GS McGeoch DJ Minson AM Pellett PE Roizman B Studdert MJ Thiry E The order Herpesvirales Arch Virol 2009 154 171 177 10.1007/s00705-008-0278-4 19066710 PMC3552636

[B24] Le Deuff RM Nicolas JL Renault T Cochennec N Experimental transmission of a herpes-like virus to axenic larvae of Pacific oyster, *Crassostrea gigas* Bull Eur Assoc Fish Pathol 1994 14 69 72

[B25] Arzul I Renault T Lipart C Davison A Evidence for interspecies transmission of oyster herpesvirus in marine bivalves J Gen Virol 2001 82 865 870 11257192 10.1099/0022-1317-82-4-865

[B26] Renault T Le Deuff RM Chollet B Cochennec N Gérard A Concomitant herpes-like virus infections in hatchery-reared larvae and nursery-cultured spat *Crassostrea gigas *and *Ostrea edulis* Dis Aquat Organ 2000 42 173 183 11104068 10.3354/dao042173

[B27] Friedman CS Estes RM Stokes NA Burge CA Hargove JS Barber BJ Elston RA Burreson EM Reece KS Herpes virus in juvenile Pacific oysters *Crassostrea gigas *from Tomales bay, California, coincides with summer mortality episodes Dis Aquat Organ 2005 63 33 41 15759798 10.3354/dao063033

[B28] Burge CA Griffin FJ Friedman CS Mortality and herpesvirus infections of the Pacific oyster *Crassostrea gigas *in Tomales Bay, California, USA Dis Aquat Organ 2006 72 31 43 17067071 10.3354/dao072031

[B29] Renault T Novoa B Viruses infecting bivalve molluscs Aquat Living Resour 2004 17 397 409 10.1051/alr:2004049

[B30] Koch R Über die Ätiologie der Tuberkulose Verhandlungen des Kongresses für Innere Medizin 1882 Erster Kongress, Wiesbaden (in German)

[B31] Hill AB The environment and the disease: association or causation? Proc R Soc Med 1965 58 295 300 14283879 10.1177/003591576505800503 PMC1898525

[B32] Evans AS Causation and disease: the Henle-Koch postulates revisited Yale J Biol Med 1976 49 175 195 782050 PMC2595276

[B33] Renault T Le Deuff RM Lipart C Delsert C Development of a PCR procedure for the detection of a herpes-like virus infecting oysters in France J Virol Methods 2000 88 41 50 10.1016/S0166-0934(00)00175-0 10921841

[B34] Council Directive 95/70/EC of 22 December 1995 introducing minimum Community measures for the control of certain diseases affecting bivalve molluscs, Official Journal Legislation 1995 332 33 39

[B35] Renault T Arzul I Herpes-like virus infections in hatchery-reared bivalve larvae in Europe: specific viral DNA detection by PCR J Fish Dis 2001 24 161 167 10.1046/j.1365-2761.2001.00282.x

[B36] Renault T Arzul I Lipart C Development and use of an internal standard for oyster herpesvirus type 1 detection by PCR J Virol Methods 2004 121 17 23 10.1016/j.jviromet.2004.05.017 15350728

[B37] Scherrer B Biostatistique 1984 Gaëtan Morin Editeur, Chicoutimi

[B38] Pinheiro JC Bates DM Mixed-effects models in S and S-plus 2004 Springer, New-York

[B39] Faraway JJ Extending the linear model with R: Generalized linear, mixed effects and nonparametric regression models 2006 Boca Raton FL, Chapman, Hall

[B40] Dohoo I Martin W Stryhn H Mc Pike Veterinary epidemiologic research 2003 Charlotettown, Canada

[B41] Thébault A Cochennec N Arzul I Renault T Establishing causal link between an infectious agent and mortalities in marine molluscan aquaculture on the example of Bonamia ostreae and Herpesvirosis in oysters: proposal of a causal grid analysis Proc 10th International Symposium on Veterinary Epidemiology and Economics 2003

[B42] Moyse C Les limites de la surveillance exhaustive (déclaration obligatoire) La Lettre de l'Infectiologue 1993 8 361 362 (in French)

[B43] Toma B Dufour B Sanaa M Benet JJ Shaw A Moutou F Louza A Epidémiologie appliquée à la lutte collective contre les maladies animales transmissibles majeures 2000 AEEMA, Maisons Alfort (in French)

[B44] Saulnier D De Decker S Haffner P Cobret L Robert M Garcia C A large-scale epidemiological study to identify bacteria pathogenic to Pacific oyster *Crassostrea gigas *and correlation between virulence and metalloprotease-like activity Microb Ecol 2010 59 787 798 10.1007/s00248-009-9620-y 20012275

[B45] Sauvage C Pépin JF Lapègue S Boudry P Renault T Ostreid herpes virus 1 infection in families of the Pacific oyster, *Crassostrea gigas*, during a summer mortality outbreak: difference in viral DNA detection and quantification using real-time PCR Virus Res 2009 142 181 187 10.1016/j.virusres.2009.02.013 19428752

[B46] Le Deuff RM Renault T Gérard A Effects of temperature on herpes-like virus detection among hatchery-reared larval Pacific oyster *Crassostrea gigas* Dis Aquat Organ 1996 24 149 157

[B47] Renault T Le Deuff RM Cochennec N Chollet B Maffart P Herpes-like viruses associated with high mortality levels in larvae and spat of Pacific oysters, *Crassostrea gigas*: A comparative study, the thermal effects on virus detection in hatchery-reared larvae, reproduction of the disease in axenic larvae Vet Res 1995 26 539 543 8581037

[B48] Burge C Judah LR Conquest LL Griffin FJ Cheney DP Suhrbier A Vadopalas B Olin PG Renault T Friedman CS Summer seed mortality of the Pacific oyster, *Crassostrea gigas *Thunberg grown in Tomales Bay, California, USA: the influence of oyster stock, planting time, pathogens, and environmental stressors J Shellfish Res 2007 26 163 172 10.2983/0730-8000(2007)26[163:SSMOTP]2.0.CO;2

[B49] Soletchnik P Le Moine O Faury N Razet D Geairon P Goulletquer P Mortalité de l'huître *Crassostrea gigas *dans le bassin de Marennes-Oléon: étude de la variabilité spatiale de son environnement et de sa biologie par un système d'informations géographiques (SIG) Aquat Living Resour 1999 12 131 143 10.1016/S0990-7440(99)80022-9

[B50] Gilad O Yun S Adkison MA Way K Willits NH Bercovier H Hedrick RP Molecular comparison of isolates of an emerging fish pathogen, koi herpesvirus, and the effect of water temperature on mortality of experimentally infected koi J Gen Virol 2003 84 2661 2668 10.1099/vir.0.19323-0 13679599

[B51] Pokorova D Vesely T Piackova V Reschova S Hulova J Current knowledge on koi herpesvirus (KHV): a review Vet Med Czech 2005 50 139 147

[B52] Van Regenmortel HV Fauquet CM Bishop DHL Virus taxonomy: classification and nomenclature of viruses Seventh report of the International Committee on Taxonomy of Viruses 2000 Academic Press, San Diego

[B53] Vigneron V Solliec G Montanié H Renault T Detection of Ostreid Herpesvirus 1 (OsHV-1) DNA in seawater by PCR: influence of water parameters in bioassays Dis Aquat Organ 2004 62 35 44 15648829 10.3354/dao062035

[B54] Bettarel Y Sime-Nganda T Amblard C Laveran H A comparison of methods for counting viruses in aquatic systems Appl Environ Microbiol 2000 66 2283 2289 10.1128/AEM.66.6.2283-2289.2000 10831400 PMC110512

[B55] Arzul I Renault T Thébault A Gérard A Detection of oyster herpesvirus DNA and proteins in asymptomatic *Crassostrea gigas *adults Virus Res 2002 84 151 160 10.1016/S0168-1702(02)00007-2 11900847

[B56] Renault T Mahy BWJ, Van Regenmortel MHV Shellfish viruses Encyclopedia of Virology 2008 Elsevier, Oxford 560 567

[B57] Moss JA Burreson EM Cordes JF Dungan CF Brown GD Wang A Wu X Reece KS Pathogens in *Crassostrea ariakensis *and other Asian oyster species: implications for non-native oyster introduction to Chesapeake Bay Dis Aquat Organ 2007 77 207 223 18062472 10.3354/dao01829

[B58] Pépin JF Riou A Renault T Rapid and sensitive detection of ostreid herpesvirus 1 in oyster samples by real-time PCR J Virol Methods 2008 149 269 276 10.1016/j.jviromet.2008.01.022 18342377

[B59] Miossec L Allain G Arzul I Francois C Garcia C Cameron AA First results of an epidemiological study on oyster (*Crassostrea gigas*) mortality events in France during summer 2008 Proc. 12th International Symposium on Veterinary Epidemiology and Economics 2009 23

[B60] Allain G Arzul I Chollet B Cobret L de Decker S Faury N Ferrand S François C Garcia C Haffner P Joly JP Michel J Miossec L Morga B Nicolas JL Omnes E Pépin JF Robert M Saulnier D Schikorski D Segarra A Tourbiez D Renault T Summer mortality outbreaks of French Pacific oysters, *Crassostrea gigas*, in 2008: research and detection of pathogens Proc. 14th EAFP International Conference, Diseases of Fish and Shellfish 2009 127

[B61] Segarra A Pépin JF Arzul I Morga B Faury N Renault T Detection and description of a particular Ostreid herpesvirus 1 genotype associated with massive mortality outbreaks of Pacific oysters, *Crassostrea gigas*, in France in 2008 Virus Res 2010 153 92 99 10.1016/j.virusres.2010.07.011 20638433

